# Transcriptome analysis reveals unique C4-like photosynthesis and oil body formation in an arachidonic acid-rich microalga *Myrmecia incisa* Reisigl H4301

**DOI:** 10.1186/1471-2164-14-396

**Published:** 2013-06-13

**Authors:** Long-Ling Ouyang, Si-Hong Chen, Yan Li, Zhi-Gang Zhou

**Affiliations:** 1 College of Aqua-life Sciences and Technology, Shanghai Ocean University, 999 Hucheng Huan Road, Pudong New District, Shanghai 201306, China

**Keywords:** *Myrmecia incisa* Reisigl H4301, 454 Pyrosequencing, C4-Like photosynthesis, Arachidonic acid, Microalga, Lipid metabolism, Oil body, Carotenoids

## Abstract

**Background:**

Arachidonic acid (ArA) is important for human health because it is one of the major components of mammalian brain membrane phospholipids. The interest in ArA inspired the search for a new sustainable source, and the green microalga *Myrmecia incisa* Reisigl H4301 has been found a potential ArA-producer due to a high content of intracellular ArA. To gain more molecular information about metabolism pathways, including the biosynthesis of ArA in the non-model microalga, a transcriptomic analysis was performed.

**Results:**

The 454 pyrosequencing generated 371,740 high-quality reads, which were assembled into 51,908 unique sequences consisting of 22,749 contigs and 29,159 singletons. A total of 11,873 unique sequences were annotated through BLAST analysis, and 3,733 were assigned to Gene Ontology (GO) categories. Kyoto Encyclopedia of Genes and Genomes (KEGG) pathway analysis uncovered a C4-like photosynthesis pathway in *M*. *incisa*. The biosynthesis pathways of lipid particularly those of ArA and triacylglycerol (TAG) were analyzed in detail, and TAG was proposed to be accumulated in oil bodies in the cytosol with the help of caleosin or oil globule-associated proteins. In addition, the carotenoid biosynthesis pathways are discussed.

**Conclusion:**

This transcriptomic analysis of *M*. *incisa* enabled a global understanding of mechanisms involved in photosynthesis, *de novo* biosynthesis of ArA, metabolism of carotenoids, and accumulation of TAG in *M. incisa*. These findings provided a molecular basis for the research and possibly economic exploitation of this ArA-rich microalga.

## Background

Arachidonic acid (ArA, 20:4^Δ5, 8, 11, 14^), an ω-6 long-chain polyunsaturated fatty acid (PUFA), is one of the major components of mammalian brain membrane phospholipids. It makes up approximately 20% of the total fatty acids along with docosahexaenoic acid (22:6ω3, DHA) in the brain [1]. In addition, ArA is an important precursor for the biosynthesis of diverse eicosanoids, such as prostaglandins, leukotrienes and thromboxanes, which play important roles in smooth muscle stimulation, platelet aggregation control and the release of histamine during anaphylactic shock and other allergic reactions [2]. ArA deficiencies have been associated with neuro-visual development disorders and other premature birth complications [3-6]. ArA is therefore considered an essential nutrient in addition to DHA during early infant development and is suggested to be added into baby formula by the Food and Agriculture Organization (FAO). Besides, carotenoids are essential nutrients and important health beneficial compounds with respect to their antioxidant properties and ability to alleviate chronic diseases [7]. Human beings are mostly incapable to synthesize carotenoids *de novo*, therefore, they rely upon diet to obtain these compounds.

ArA can be commercially obtained from marine fish oil, animal tissues and fungi [8]. Interest in ArA and other long-chain PUFAs inspired the search for new PUFA sources. *Myrmecia incisa* Reisigl H4301, a coccoid green microalga species of Trebouxiophyceae [9], has recently been reported to accumulate an unprecedentedly high amount of ArA-rich triacylglycerols (TAG) in cytoplasmic lipid bodies [10]. When *M*. *incisa* was cultured under nitrogen starvation for 27 d, its ArA content increased from 1.9% to 7.0% of dry weight (DW) biomass and 76% of the intracellular ArA accumulated in the form of neutral lipids [10]. In addition, carotenoids content increased as well under nitrogen starvation in *Parietochloris incisa* (synonym with *M*. *incisa*[11,12]) [13]. Thus, *M*. *incisa* may be a potential resource for both ArA and carotenoids exploitation.

There are two pathways proposed in microalgae for ArA biosynthesis [14-16]. The understanding of ArA biosynthesis pathway in *M. incisa*, thus, is first of all for the improvement of ArA level. Therefore, genes encoding fatty acid desaturases and elongase of this microalga have been characterized, and some routes were determined by heterologous expression of these genes in model organisms [10,17-19]. Carotenogenesis pathways in microalgae are similar to those in higher plants on one hand, and possess microalgae-specific features on the other hand [20]. Some common carotenogenesis genes have been identified in microalgae [20-22], whereas the molecular information for carotenoids biosynthesis in *M*. *incisa* is rather limited [10,11].

Expressed sequence tag (EST) analysis is the primary tool for novel gene discovery, particularly in non-model organisms for which full genome sequencing is not economically feasible. By using Sanger sequencing, a cDNA library from *M. incisa* generated only 595 unique sequences from 1,854 readable ESTs with little information of interest [11]. In contrast to this sequencing method, next-generation sequencing (NGS) technologies have led to a revolution in genomics and genetics and provided cheaper and faster delivery of sequencing information [23,24]. Currently, the newest 454 pyrosequencing platform, GS FLX Titanium, is one of the most widely used NGS technologies for the *de novo* sequencing and analysis of transcriptome in both the model organism of microalga, *Chlamydomonas reinhardtii*[25] and non-model ones including *Alexandrium tamarense* CCMP1598 [26], *Botryococcus braunii* BOT-88-2 [27], *Botryococcus braunii* BOT-22 [28], *Dunaliella tertiolecta* UTEX LB 999 [29] and *Oxyrrhis marina*[30].

In this paper, the 454 pyrosequencing method was used to gain more insight into the *M*. *incisa* metabolism pathways. Global transcriptome analysis revealed a C4-like photosynthesis pathway and a detailed lipid metabolism pathway. Furthermore, the identification of sequences coding for caleosin and oil globule-associated protein in the transcriptome of *M. incisa* provided evidence for accumulation of TAG in the form of oil body. Finally, the disclosure of the genes involved in the biosynthesis of carotenoids in *M*. *incisa* may favor the commercial exploitation of this algal resource in addition to ArA production.

## Results and discussion

### 454 pyrosequencing and *de novo* assembly

**Figure 1:**
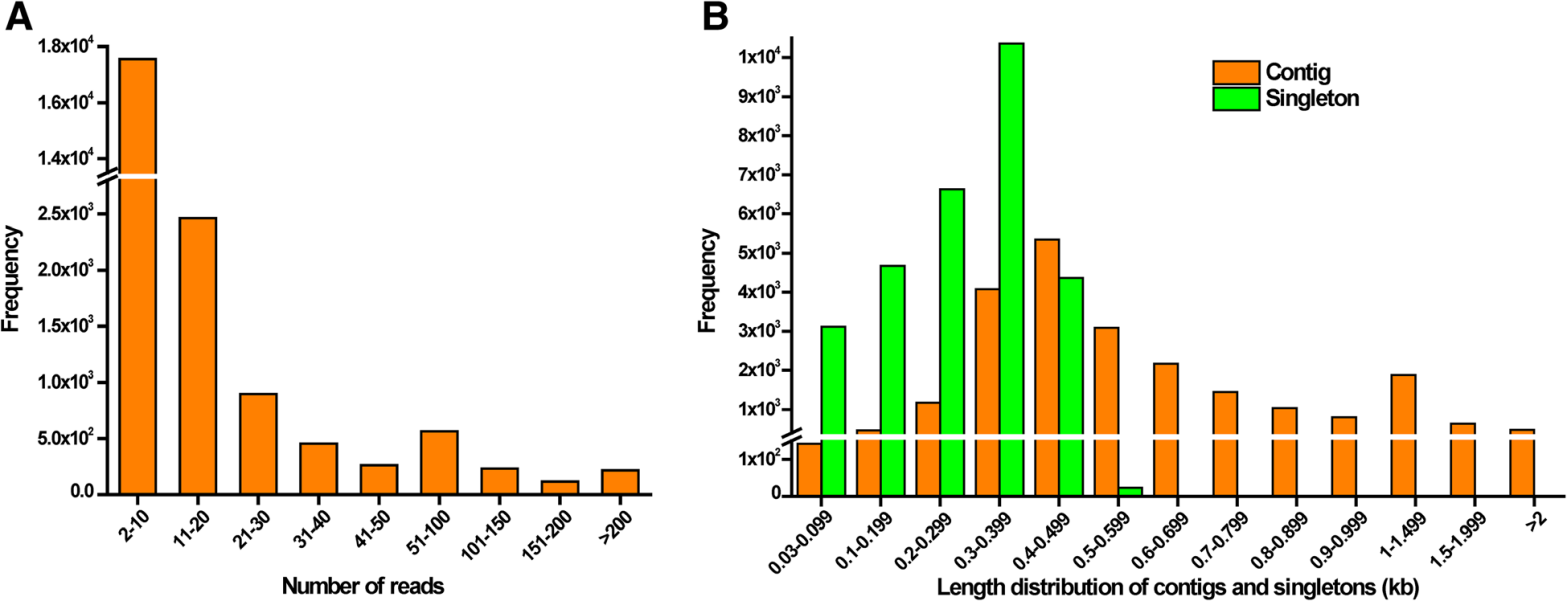
**Overview of the assembly of the *M*. *incisa *H4301 transcriptome.** Distribution of number of reads assembled into contig (**A**) and the size distribution of the contigs and singletons (**B**). The unit of the x-axis in Figure 1A is number of reads and in Figure 1B is kilobases (kb).

In total, 381,804 reads (minimal size > 29 bp) with an average length of 332 bp were generated from one pyrosequencing run. Filtering (e.g., removal of primers and poly A tail sequences) of the raw sequences resulted in 371,740 high-quality (HQ) reads with an average length of 321 nucleotides, generating 119 Mb corresponding to 97.36% of the raw sequences (Table 1). After clustering and assembling using CAP3 clustering tools [31], these reads were assembled into 51,908 unique sequences consisting of 22,749 contiguous sequences (contigs) and 29,159 singletons (i.e., reads not assembled into contigs) (Table 1). Approximately 77% of the total contigs comprised 2 to 10 reads (Figure 1A). The average length of the total contigs, including 55.03% that ranged from 300 to 599 bp, was 642 ± 438 bp, while the average length of singletons, including 89.25% that ranged from 100 to 499 bp, was 299 ± 98 bp (Figure 1B). In comparison with transcriptomes sequenced by 454 pyrosequencing from other microalgae, the HQ percentage of the *M*. *incisa* reads was nearly the same as that for *D*. *tertiolecta* but higher than that for *Oxyrrhis marina* (Table 2). In addition, the average length of the *M*. *incisa* reads was shorter than that of *D*. *tertiolecta* but longer than that of *B*. *braunii* BOT-88-2, *B*. *braunii* BOT-22 and *Oxyrrhis marina* (Table 2). This finding indicates that the sequencing data are creditable for further analysis. All of the HQ reads are available in the Sequence Read Archive (SRA) under the accession number SRA061977.

**Table 1:** **Summary of the transcriptome characteristics of *M. incisa***

	**Total**	**Contigs**	**Singletons**
Number of raw reads	381,804		
Average nucleotide length of raw reads (bp)	332		
Number of HQ reads	371,740		
Average nucleotide length of HQ reads (bp)	321		
Number of unique sequences	51,908	22,749	29,159
Average nucleotide length of unique sequences (bp)		642 ± 438	299 ± 98
Unique sequences with BLAST matches	11,873	7,361	4,512
Unique sequences annotated with GO and KEGG pathways	3,733	2,199	1,534
Unique sequences assigned with EC numbers	2,557	2,019	538

**Table 2:** **454 pyrosequencing comparison between *M. incisa *and other microalgae**

**Species**	**Number of raw reads**	**Number of HQ reads**	**Percentage of HQ reads**	**Average length of HQ reads (bp)**	**Number of contigs**	**Number of unique sequences**	**Reference**
*Myrmecia incisa* H4301	381,804	371,740	97.36	321	22,749	51,908	This study
*Alexandrium tamarense*	NA^a^	1,073,382	NA	NA	NA	40,029	[26]
*Botryococcus braunii* BOT-88-2	NA	185,936	NA	217	NA	29,038	[27]
*Botryococcus braunii* BOT-22	NA	209,429	NA	202	NA	27,427	[28]
*Dunaliella tertiolecta* UTEX LB 999	1,385,389	1,365,353	98.55	400	33,307	409,789	[29]
*Oxyrrhis marina*	299,081	238,240	79.66	228	7,398	50,994	[30]

^a^ Not available.

### Functional annotation

A total of 7,361 contigs (32.36% of total contigs) and 4,512 singletons (15.47% of total singletons) had significant BLAST matches from selection of the best hits against Swiss-Prot, GenBank database and JGI Genome Portal at an e-value ≤ 10^-3^ (Table 1). The percent of annotated contigs was consistent with the 20% to 40% of the values previously reported for *de novo* eukaryotic transcriptome assemblies [32-34].

**Figure 2:**
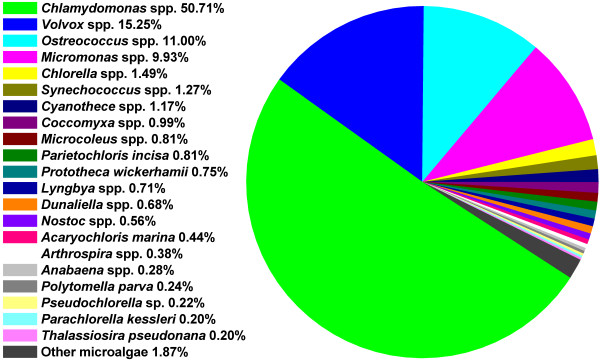
**Distribution of microalgal species to which the unique sequences from *M. incisa* H4301 transcriptome were homologous by BLAST searches.**

Analysis of the 11,873 BLAST-matched unique sequences suggested that there is less molecular information available for microalgae in comparison with other organisms in public databases as only approximately 42% of the BLAST-matched unique sequences were homologous to microalgae. However, unique sequences matched to genes from green microalgae accounted for 92% approximately, demonstrating a distinct microalgae character for the transcriptome of *M*. *incisa* (Figure 2). The matched green microalgal species were mainly *Chlamydomonas reinhardtii* (Chlorophyceae), *Volvox carteri* (Chlorophyceae), *Ostreococcus* spp. (Mamiellophyceae) and *Micromonas* spp. (Mamiellophyceae) (Figure 2), of which *Chlamydomonas reinhardtii* is a model organism with a sequenced genome that has been the focus of most physiological, molecular and genetic studies [35]. The sequenced genomes of *Ostreococcus tauri*, *Micromonas* sp. RCC299 and *Micromonas* sp. CCMP1545 have offered valuable information for eukaryotic genome evolution and unusual photosynthesis apparatuses such as C4-like photosynthesis [36,37].

**Figure 3:**
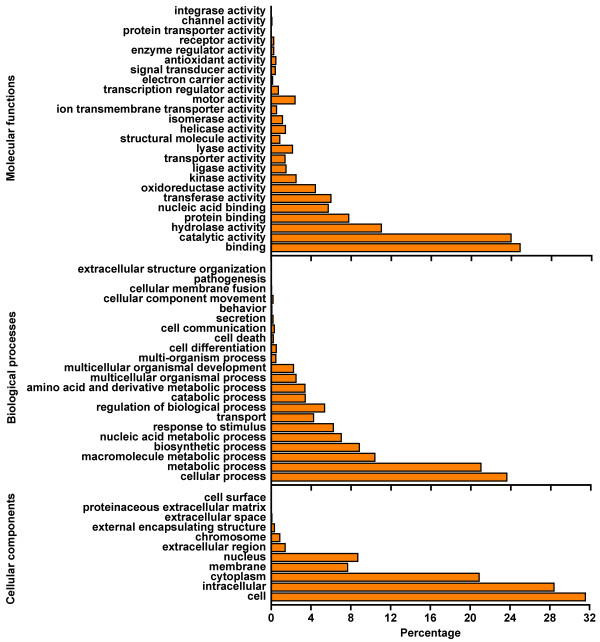
**Gene ontology (GO) functional analysis of unique sequences from *M*. *incisa *H4301 transcriptome.** Unique sequences were assigned to three categories: molecular functions, biological processes and cellular components.

Of the 11,873 unique sequences with BLAST matches, 31.44% (2,199 contigs and 1,534 singletons) were annotated with GO terms, and 68.50% of the GO annotated unique sequences (2,019 contigs and 538 singletons) were assigned EC numbers (Table 1). One unique sequence may be assigned to several GO categories, and the distribution of the most abundant GO terms for molecular functions, biological processes and cellular components is presented in Figure 3. GO annotation for cellular component and biological process categories highlighted the dominance of unique sequences associated with metabolic processes in cell, especially in cytoplasm, with fewer unique sequences involved with cell differentiation (0.5%), communication (0.31%) and cellular component movement (0.17%). The latter is consistent with the unicellular morphology without any flagella during the vegetative growth of *M*. *incisa*[11]. The molecular functional distribution indicated the top two assigned categories are binding processes (about 26%) and catalytic activity (about 24%). Besides, no pathogenesis process occurred in this microalga reflecting to some extent the axenic culture of *M*. *incisa* in this study.

However, this analysis only provided a general concept for the function of these unique sequences but no assignment for the metabolic pathways that they are involved in. For further detailed understanding, unique sequences were then analyzed for KO identifiers using the KEGG database.

### Pathway assignment by KEGG

Functional classification and pathway assignment was performed using KEGG with the annotated unique sequences. As shown in Additional file 1, the relatively complete pathways that the unique sequences were assigned to were related to translation, replication and repair, amino acid metabolism and energy and substance metabolism including glycolysis, the TCA cycle, pyruvate metabolism, photosynthesis, carbon fixation, starch and sucrose metabolism, arachidonic acid metabolism, glycerolipid metabolism and carotenoid biosynthesis. Particular attention focused on the biosynthesis pathways of ArA-rich TAG and carotenoids because *M*. *incisa* had the potential to accumulate these compounds, particularly under nitrogen starvation [10,13]. Interestingly, the assigned photosynthesis pathway suggested that *M*. *incisa* possessed some unique features for the carbon concentrating mechanism (CCM) as described below.

### The C4-like photosynthesis pathway in *M*. *incisa*

The central carboxylation enzyme for CO_2_ fixation during photosynthesis is named ribulose-1, 5- bisphosphate carboxylase/oxygenase (RubisCO), but its affinity to CO_2_ is low [38]. To solve this problem, some plants, including several microalgae, such as *Phaeodactylum tricornutum*[39], *Thalassiosira pseudonana*[40], *Ostreococcus tauri*[36] and *Micromonas* sp. [37], have developed a C4-like photosynthesis pathway in addition to the biophysical CCM [bicarbonate transport and external or intercellular carbonic anhydrase (CA, EC 4.2.1.1)].

Based on the transcriptome analysis, it is interesting to find that *M*. *incisa* possibly possesses a C4-like photosynthesis pathway during the CCM. Several unique sequences encoding phosphoenolpyruvate carboxylase (PEPC, EC 4.1.1.31), phosphoenolpyruvate carboxykinase (PPCK, EC 4.1.1.32) and pyruvate orthophosphate dikinase (PPDK, EC 2.7.9.1) were identified (Additional file 2). Each of these enzymes is necessary for C4-metabolism. Three unique sequences encoding PEPC have been identified, including one that is 3,932 bp long with 71% amino acid identity with *Coccomyxa subellipsoidea* C-169 [GenBank: EIE27459]. In addition, the putative protein sequence of this identified unique sequence revealed a PEP binding site, an HCO_3_^-^ trapping site and a C-terminal tetrapeptide QNTG [41,42]. Subcellular localization analysis predicted that this PEPC possesses neither a signal nor transit peptide (Additional file 2), suggesting that it may function within the cytosol to produce oxaloacetic acid (OAA).

On the one hand, OAA is assumed to be transaminated to aspartate (Asp) by Asp aminotransferase (AAT, EC 2.6.1.1) [43]. In fact, AAT can catalyze the reversible transfer of OAA and glutamate to Asp and α-ketoglutarate. Eight unique sequences were identified to encode AAT, and one was cytoplasmic and another was mitochondrial (Additional file 2). The transaminated product in the cytosol, Asp, is subsequently imported into mitochondria via an Asp-glutamate carrier, which is important in malate/Asp cycle [44]. One unique sequence in this transcriptome has been identified to have 53% identity with a mitochondrial isoform [GenBank: BAC84529] from *Oryza sativa*. Once Asp is transported into mitochondria, the mitochondrial AAT functions to regenerate OAA, which is then decarboxylated to PEP by a mitochondria-localized PPCK. Afterwards, the PEP may be converted to pyruvate with the concomitant phosphorylation of ADP to ATP by pyruvate kinase (PK, EC 2.7.1.40) catalysis. A NAD-dependent malate dehydrogenase (MDH-NAD^+^, EC 1.1.1.37) with a predicted mitochondrial transit peptide and its downstream enzyme NAD-dependent malic enzyme (ME-NAD^+^, EC 1.1.1.38), which has 46% identity with the mitochondrial ME-NAD^+^ [GenBank: XP_002265765] from *Vitis vinifera*, were identified (Additional file 2). The resulting pyruvate from these two OAA decarboxylation pathways could participate in the TCA cycle.

**Figure 4:**
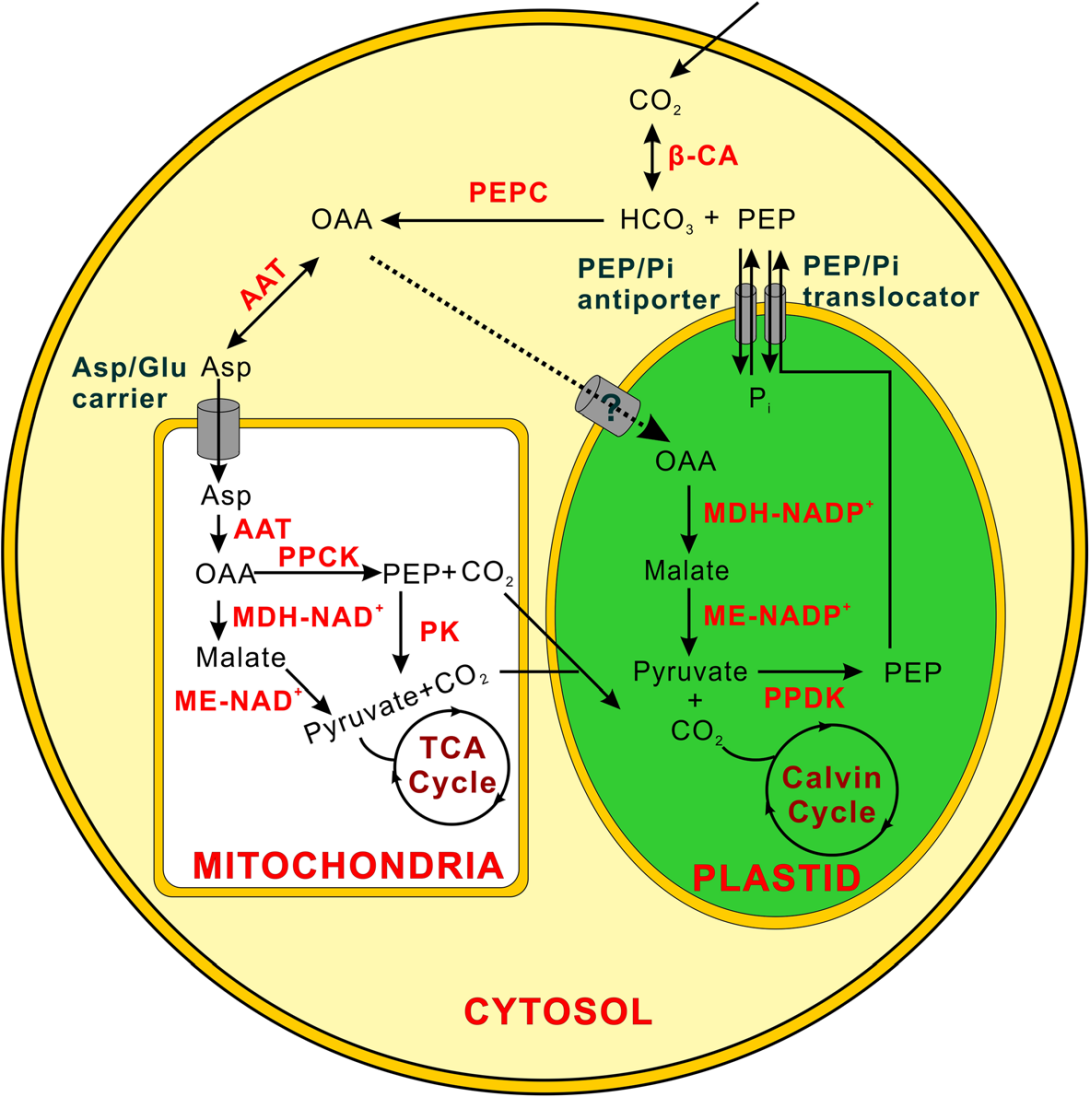
**Model of the carbon concentration mechanisms in *M*. *incisa *H4301 based on the transcriptome.** The dashed line indicates the routes that was not represented in this transcriptome. Abbreviations are listed as follows: PEP, phosphoenolpyruvate; AAT, aspartate aminotransferase; OAA, oxaloacetic acid; Asp, aspartate; Pi, inorganic phosphate; CA, carbonic anhydrase; MDH-NADP^+^, NADP-dependant malate dehydrogenase; MDH-NAD^+^, NAD-dependant malate dehydrogenase; ME-NADP^+^, NADP-dependant malic enzyme; ME-NAD^+^, NAD-dependant malic enzyme; PEPC, phosphoenolpyruvate carboxylase; PPCK, phosphoenolpyruvate carboxykinase; PK, pyruvate kinase; PPDK, pyruvate-phosphate dikinase; AAT, aspartate aminotransferase.

On the other hand, OAA is assumed to be reduced to malate by NAPD-dependent MDH (MDH-NADP^+^, EC 1.1.1.82), which targets chloroplasts [45]. Although the mechanism of the OAA shuttle from the cytosol to chloroplast is unknown [39], a unique sequence encoding chloroplast-localized MDH-NADP^+^ was identified in *M*. *incisa*. In addition, a putative NADP-dependent ME-coding unique sequence (ME-NADP^+^, EC 1.1.1.40) with 52% identity with a chloroplastic isoform [GenBank: XP_003546557] from *Glycine max* was annotated (Additional file 2). Dehydrogenated malate from OAA is decarboxylated by ME-NADP^+^ to generate two different products CO_2_ and pyruvate. The former product is fixed by RubisCO along with the Calvin cycle, and the latter is converted by PPDK to PEP, which is then transported to the cytosol as the substrate of PEPC by PEP/Pi translocators. There are two putative chloroplast-localized PEP/Pi translocators, and they have 74 and 52% identity with the chloroplastic isoforms from *Brachypodium distachyon* [GenBank: XP_003577954] and *Coccomyxa subellipsoidea* C-169 [GenBank: EIE22024] (Additional file 2), respectively. In addition, a unique sequence encoding a PEP/Pi antiporter was identified, demonstrating that there must be a PEP and Pi exchange between chloroplast and the cytosol in *M*. *incisa* (Figure 4). The predicted subcellular localization of PPDK, which had 96% homology with that from *Methylobacterium radiotolerans* JCM 2831 [GenBank: YP_001755475], is unknown due to an incomplete sequence. However, the identified PEP/Pi translocator (antiporter) implied that there should be one chloroplastic PPDK in *M*. *incisa*.

In summary, the identification of the crucial enzymes of C4-like photosynthesis, such as PEPC, PPCK and PPDK, and the transporters highlighted the CCM in *M*. *incisa*. It possesses both CA (Additional file 2) and a C4-like photosynthesis pathway, thus enabling it to adapt to a low CO_2_ level habitat. It is another microalga in Chlorophyta with a C4-like CCM besides *Ostreococcus tauri*[36] and *Micromonas* sp. [37].

### *De novo* synthesis of fatty acids

**Figure 5:**
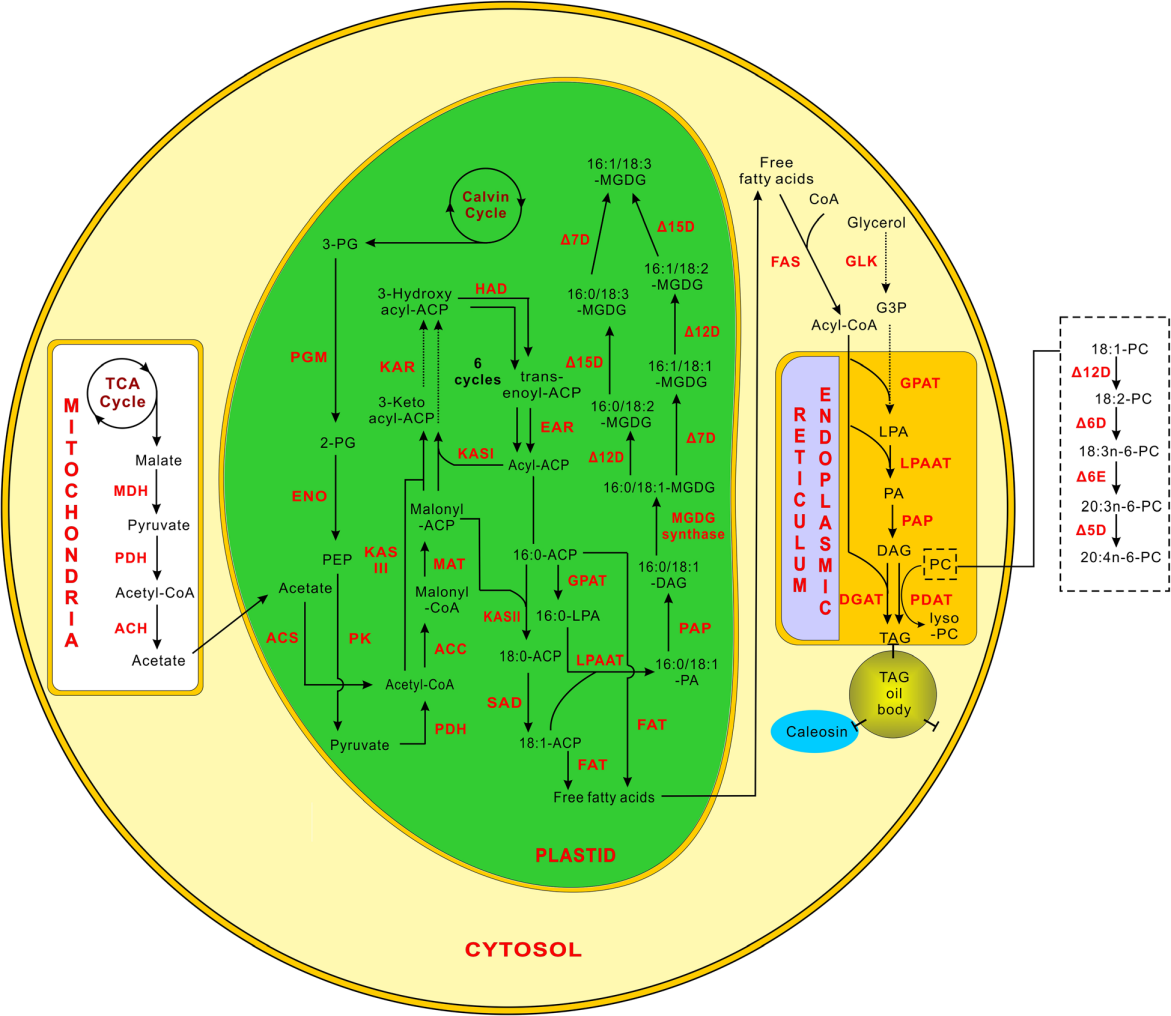
**Fatty acid biosynthesis and their accumulation in *M*. *incisa *H4301 based on the transcriptome.** The dashed lines indicate the routes that were not represented in this transcriptome. Abbreviations are listed as follows: MDH, malate dehydrogenase; PDH, pyruvate dehydrogenase/decarboxylase; ACH, acetyl-CoA hydrolase; ACS, acetyl-CoA synthetase; KAS, 3-ketoacyl-ACP synthase; HAD, 3-hydroxyacyl-ACP dehydratase; KAR, 3-oxoacyl ACP reductase; MAT, malonyl-CoA:ACP transacylase; ACC, acetyl-CoA carboxylase; EAR, enoyl-ACP reductase; SAD, stearoyl-ACP desaturase; FAT, fatty acid thioesterase; PAP, phosphatidic acid phosphatase; MGDG, monogalactosyldiacylglycerol; Δ9D, Δ9 desaturase; Δ12D, Δ12 desaturase; Δ15D, Δ15 desaturase; Δ7D, Δ7 desaturase; Δ5D, Δ5 desaturase; Δ6E, Δ6 elongase; FAS, fatty acid synthase; GLK, glycerol kinase; GPAT, glycerol 3-phosphate acyltransferase; LPAAT, 1-acyl-sn-glycerol-3-phosphate acyltransferase; DGAT, diacylglycerol acyltransferase; PDAT, phopholipid:diacylglycerol acyltransferase.

Global lipid biosynthesis pathways have been well known in many higher plants [46], whereas fatty acid biosynthesis and accumulation in microalgae have not been well studied because the genetic background of microalgae is limited. High-throughput sequencing, however, enabled us to understand lipid metabolism in *B*. *braunii*[27], *D. tertiolecta*[29] and *M*. *incisa* (Figure 5).

In all plants, palmitic (16:0) and oleic acid (18:1) are almost exclusively *de novo* synthesized in chloroplasts via the key precursor acetyl-coenzyme A (acetyl-CoA) [47]. There are two possible processes for supplying acetyl-CoA: one is directly from plastid pyruvate through the catalysis of pyruvate dehydrogenase/decarboxylase (PDH, EC 1.2.4.1), and the other is an indirect regeneration from the imported mitochondrial acetate through the catalysis of acetyl-CoA synthetase (ACS, EC 6.2.1.1) in plastid. The generation of acetate in mitochondria begins as pyruvate with acetyl-CoA as an intermediate and is sequentially catalyzed by mitochondrial PDH and acetyl-CoA hydrolase (ACH, EC 3.1.2.20) [47]. Based on transcriptome analysis, unique sequences encoding chloroplastic and mitochondrial PDH have been successfully identified (Figure 5). In addition, the putative mitochondrial ACH and chloroplastic ACS were identified (Additional file 2), demonstrating consistency with the description by Harwood [47].

The carbon chain condensation reaction begins with acetyl-CoA via the initial catalysis by acetyl-CoA carboxylase (ACC, EC 6.4.1.2). The generated malonyl-CoA is subsequently converted into malonyl-acyl carrier protein (malonyl-ACP) by malonyl-CoA:ACP transacylase (MAT, EC 2.3.1.39), and the product, malonyl-ACP, participates in whole fatty acid synthesis as an all-important source of 2C units. To complete the following series of condensation reactions, 3-ketoacyl-ACP synthase (KAS), 3-oxoacyl ACP reductase (KAR, EC 1.1.1.100), 3-hydroxyoctanoyl-ACP dehydratase (HAD, EC 4.2.1.59) and enoyl-ACP reductase (EAR, EC 1.3.1.9) are involved. During this course, three condensing enzymes, KASI, KASII and KASIII, play different roles [47]. It has been reported that the subunits of KASI and KASII dimers are encoded by two closely related plastid genes [48]. However, KASI extends the carbon chain length from 4C to 16C during six rounds of 2C elongation, whereas KASII elongates palmitate (16:0) to stearate (18:0). KASIII, which is distinct from KASI and KASII and is found in all plants [49], catalyzes the initial condensation reaction using acetyl-CoA rather than acetyl-ACP as a substrate for plant fatty acid synthesis (Figure 5). In *M. incisa*, at least one putative unique sequence of each type of KAS was identified, suggesting that carbon chain condensation reactions in this alga are the same as that in most plants.

After the formation of ACP-bound 18:0, the first double bond is introduced via the catalysis of stearoyl-ACP desaturase (SAD, EC 1.14.19.2). This enzyme, which is present in the plastids of higher plants and algae, is the only soluble Δ9 desaturase [50,51]. In this transcriptome, one SAD-coding unique sequence with 65% homology with plastid acyl-ACP desaturase from *Coccomyxa subellipsoidea* C-169 [GenBank: EIE22226] was identified. Products of fatty acid *de novo* biosynthesis, ACP-bound 16:0 and 18:1^Δ9^ may subsequently constitute the biomembrane as a structural component or be exported into endoplasmic reticulum (ER) for the biosynthesis of ArA as a storage lipid (Figure 5).

### Biosynthesis of ArA and TAG

In eukaryotic algae, most of the desaturation of fatty acids is catalyzed by acyl-lipid desaturases rather than by acyl-ACP desaturase. In chloroplasts, glycolipids, which mostly include monogalactosyldiacylglycerol (MGDG), serve as substrates for chloroplastic Δ12 (Δ12D, EC 1.14.19.6) and ω3 desaturases. In this study, several unique sequences encoding putative chloroplastic Δ12D and ω3 desaturases, which the latter has been confirmed to be Δ15 desaturase (Δ15D, EC 1.14.19.-) [18], were identified. In addition, a full-length cDNA sequence of MGDG-specific palmitate Δ7 desaturase (Δ7D, EC 1.14.99.-), which is involved in desaturation leading to the formation of 16:1^Δ7^, was also identified in this transcriptome. The biosynthesis of PUFAs taking place in the ER is similar to that in chloroplasts with the exception of those utilizing phospholipids, i.e., phosphatidylcholine (PC), as substrates (Figure 5). During the culture of *M. incisa*, a comparison of fatty acid content between complete and nitrogen-deficient medium suggested a possible biosynthesis pathway for ArA, which begins with 18:1^Δ9^ with 18:2^Δ9, 12^, 18:3^Δ6, 9, 12^ and 20:3^Δ8, 11, 14^ as successive intermediates. In this process, the ER-harbored Δ12D, Δ6 desaturase (Δ6D, EC 1.14.19.3), Δ6 elongase (Δ6E, EC 6.21.3.-) and Δ5 desaturase (Δ5D, EC1.14.99.-) were involved; the genes encoding these enzymes have been cloned as well [10]. The result of this study and the cloned genes again confirmed the presence of an ArA biosynthesis pathway in this microalga. Moreover, three other Δ5D-coding unique sequences were identified in this transcriptome (Additional file 2). Although the subcellular localization of the other Δ5D was unpredictable due to incomplete sequences, these sequences provided further information for the investigation of ArA and other PUFA biosynthesis. The capacity of PUFAs may enhance the fluidity of the phospholipid membrane, which makes it possible for *M*. *incisa* to withstand chilling or cold stress [52].

A previous study has reported that most of the intracellular ArA was accumulated in the form of TAG in *M. incisa*, particularly during nitrogen starvation [10]. During the accumulation process, glycerol-3-phosphate (G-3-P) and acyl-CoA serve as primary substrates in the Kennedy pathway (Figure 5). One of the substrates, G-3-P, is generated by glycolysis or the catalysis of free glycerol by glycerol kinase (GLK, EC 2.7.1.30). However, sequence coding for GLK was not identified in this study. The other substrate, acyl-CoA, is generated via the esterification of free fatty acids exported from chloroplasts with CoA. These two substances are acylated by a series of acyltransferases including the initial glycerol 3-phosphate acyltransferase (GPAT, E.C: 2.3.1.15). This enzyme catalyzes the acylation of the sn-1 position of G-3-P to produce 1-acyl-sn-glycerol-3-phosphate (LPA) [53]. The sn-2 position of LPA is acylated by 1-acyl-sn-glycerol-3-phosphate acyltransferase (LPAAT, EC 2.3.1.51) to generate phosphatidic acid (PA). Unique GPAT- and LPAAT-coding sequences were identified in the *M*. *incisa* transcriptome. Subcellular localization prediction analysis demonstrated that GPAT might target chloroplasts, whereas the localization of LPAAT was unknown due to the lack of a complete sequence (Additional file 2). PA is subsequently converted into either diacylglycerol (DAG) by phosphatidic acid phosphatase (PAP, EC 3.1.3.4) or cytidine diphosphate-diacylglycerol (CDP-DAG) by phosphatidate cytidylyltransferase (EC 2.7.7.41), and these two types of lipids alternately generate various phospholipids. The storage lipid, TAG, is either formed via an acyl-CoA-dependent reaction or an acyl-CoA-independent reaction, and both of them use DAG as an acyl-acceptor. The acyl-CoA-dependent reaction, which is catalyzed by diacylglycerol acyltransferase (DGAT, EC 2.3.1.20), uses fatty acid acyl-CoAs as acyl donors, whereas the acyl-CoA-independent reaction is mainly catalyzed by a phospholipid:diacylglycerol acyltransferase (PDAT, EC 2.3.1.158) uses PC as acyl donors instead. DGAT has two homologs, DGAT1 and DGAT2, and their coding genes belong to the cholesterol acyltransferase gene family and the monoacylglycerol acyltransferase gene family, respectively. In *M*. *incisa*, there are five unique sequences encoding DGAT, three were identified as DGAT1, and the rest were identified as DGAT2 (Additional file 2). In algae, membrane lipids, including photosynthetic ones, rapidly degrade with the accumulation of cytosolic TAG-enriched lipid bodies when cells encounter stress [54]. If a PDAT orthologue was identified, particularly in chloroplasts, then it is conceivable that PC or even galactolipids may be utilized as acyl donors in TAG synthesis [54]. Three unique sequences coding for PDAT in this transcriptome were all partial sequences; thus, subcellular localization could not be predicted. However, this finding has already demonstrated that *M*. *incisa* is able to convert these membrane lipids into storage TAG.

*M*. *incisa* has been reported to be able to accumulate TAG in densely packed oil bodies [10]. Oil bodies in higher plant seeds are the most prominent and well studied [55-57]. They are spherical organelles consisting of neutral lipids enclosed by a semi-layer membrane of phospholipids coated with proteins (Figure 5). Thus far, only two proteins associated with oil bodies have been well described: oleosin and caleosin. Oleosins are mostly found in higher plants and are thought to be important for oil body stabilization in the cytosol, while caleosins are thought to be ubiquitous in higher plants and algae and play an important role particularly in oil body degradation during seed germination [58,59]. In *Chlamydomonas reinhardtii*, oleosin-like and caleosin-like genes were identified in the genome, whereas the relevant proteins were not identified by proteomic analysis [60]. Two unique sequences encoding caleosin with an EF-hand domain were identified, whereas none of the oleosin coding unique sequences were identified in this ArA-rich microalga (Additional file 2), thus implying this caleosin may play an important role in oil body formation. In addition, two unique sequences encoding oil globule-associated proteins were identified (Additional file 2), which may also be related to oil body formation. However, the functional characteristics of these two genes coding for caleosin and oil globule-associated protein need further studies.

### TAG degradation

Degradation of the storage lipid TAG plays an important role in the reconstruction of the cytoplasmic membranes of organisms grown under favorable conditions. The complete TAG degradation process includes a series of hydrolysis reactions via lipase [61]. This reaction begins with the release of fatty acids from the sn-3 or sn-1 positions of TAG to form DAG by triacylglycerol lipase (TAGL, EC 3.1.1.3). DAG is subsequently hydrolyzed into a fatty acid and 2-monoacylglycerol (2-MAG) via the catalysis of diacylglycerol lipase (DAGL, EC 3.1.1.-). Isomerization of the latter product to 1(3)-monoacyl-sn-glycerols occurs to some extent, and these may be completely degraded to glycerol and free fatty acids by monoacylglycerol lipase (MAGL, EC 3.1.1.23) (Additional file 3). The α- and β-type DAGL and TAGL-coding unique sequences were identified in this transcriptome (Additional file 2). It is worth mentioning that caleosin, as indicated above, may also play a role in TAG degradation in *M. incisa* according to previous reports about seed development [59].

In summary, all of the identified enzymes described above provide evidence for a better global understanding of the lipid metabolism process in *M. incisa* (Figure 5 and Additional file 3), though further study is needed to confirm their functional characteristics.

### Biosynthesis of carotenoids

In the past two decades, great advancements have been made in studies of carotenoid metabolism in higher plants and algae [62]. The entire biosynthesis process can be divided into the following major steps: (1) isopentenyl diphosphate (IPP) synthesis; (2) enzymatic phytoene formation reactions; (3) the desaturation (dehydrogenation) of phytoene to lycopene; (4) cyclization and the formation of α- and β-carotenes; and (5) the synthesis of carotenes derivatives [20].

There are two suggested pathways for IPP synthesis: the acetate-mevalonate pathway beginning with acetate in cytosol and the phosphoglyceraldehyde-pyruvate pathway beginning with pyruvate in chloroplasts [20]. There are no identified unique sequences encoding mevalonate kinase (MVK, EC 2.7.1.36), phosphomevalonate kinase (PMVK, EC 2.7.4.2) or mevalonate pyrophosphate decarboxylase (MPD, EC 4.1.1.33), which are all required for the former pathway. In contrast, most of enzyme-coding genes involved in the latter pathway have been identified, and they encode 1-deoxy-D-xylulose 5-phosphate synthase (DXS, EC 2.2.1.7), 1-deoxy-D-xylulose 5-phosphate reductoisomerase (DXR, EC 1.1.1.267), 4-(cytidine 5’-diphospho)-2-C-methyl-D-erythritol kinase (CMK, EC 2.7.1.148), 2-C-methyl-D-erythritol 2,4-cyclodiphosphate synthase (MCS, EC 4.6.1.12) and 1-hydroxy-2-methyl-2-(E)-butenyl 4-diphosphate synthase (HDS, EC 1.17.4.3). However, several unique sequences encoding the rest of the enzymes, including 2-C-methyl-D-erythritol 4-phosphate cytidyltransferase (CMS, EC 2.7.7.60) and 1-hydroxy-2-methyl-2-(E)-butenyl 4-diphosphate reductase (HDR, EC 1.17.1.2) in this pathway, were unfortunately missed (Additional file 4). It appears that *M*. *incisa* may utilize glucose as the sole carbon source to synthesize carotenoids, which is the same as that in *Chlorella* and *Scenedesmus*[63].

Products generated from the first step include IPP and dimethylallyl diphosphate (DMAPP), which may be interconverted by the catalysis of IPP isomerase (IPPi, EC 5.3.3.2). The condensation of three IPP molecules and one DMAPP molecule (Additional file 4) is catalyzed by geranyl diphosphate synthase (GPPS, EC 2.5.1.1), farnesyl diphosphate synthase (FPPS, EC 2.5.1.10) and geranyl-geranyl diphosphate synthase (GGPPS, EC 2.5.1.29) in a step-by-step manner to generate geranyl-geranyl diphosphate (GGPP). In addition to being the precursor for carotenoids, GGPP is also the precursor of several other groups of metabolites including chlorophylls, ubiquinones and tocopherols. Therefore, following the condensation of two molecules of GGPP by phytoene synthase (PSY, EC 2.5.1.32) to form phytoene is a committed step in carotenoid biosynthesis. Phytoene subsequently undergoes four steps to form lycopene by the sequential catalysis of phytoene desaturase (PDS, EC 1.3.99.29), ζ-carotene desaturase (ZDS, EC 1.3.5.6), ζ-carotene isomerase (ZISO, EC 5.2.1.12) and carotenoid isomerase (CRTISO, EC 5.2.1.13). In *M. incisa*, all of the enzymes except ZISO that are described in the lycopene formation process were successfully identified (Additional file 2).

Lycopene is an important intermediate in the biosynthesis of carotenoids, including β-carotene, which is responsible for yellow, orange or red pigments, photosynthesis and photo-protection in photosynthetic organisms. The cyclization of the lycopene catalyzed by lycopene ϵ- and β-cyclases is a critical branch-point in carotenoid biosynthesis [64]. In one branch, a single enzyme, lycopene β-cyclase (β-CYC, EC 5.5.1.19), introduces a β-ring at both ends of lycopene to form β-carotene in a sequential two-step reaction, while in the other branch, lycopene introduces one ϵ- and one β-ring at each end via ϵ-CYC (EC 5.5.1.18) followed by β-CYC to form α-carotene. Unique sequences encoding ϵ-CYC and β-CYC were identified (Additional file 2), suggesting that the biosynthesis pathway of β-carotene and α-carotene is present in *M. incisa*. β-Carotene can be hydroxylated by β-carotene hydroxylase (BCH, EC 1.14.13.129) in a two-step reaction to zeaxanthin with β-cryptoxanthin as an intermediate. In green tissues, zeaxanthin can be epoxidized to violaxanthin, and a set of light- and dark-controlled reactions known as the xanthophyll cycle rapidly optimizes the violaxanthin and zeaxanthin concentrations in the cell via the action of zeaxanthin epoxidase (ZEP, EC 1.14.13.90) and violaxanthin de-epoxidase (VDE, EC 1.10.99.3), respectively [65]. In this transcriptome, unique sequences that encode BCH, ZEP and VDE were identified (Additional file 2). *M*. *incisa* is able to biosynthesize high-value carotenoids including, at the least, the α- and β-carotenes and zeaxanthin and violaxanthin. β-carotene is a precursor for vitamin A biosynthesis, which is important for growth and maintenance of the immune system and good vision [66,67], and zeaxanthin and violaxanthin are usually used as food colourant. Due to this, further exploitation of this ArA-rich green microalga could be valuable.

## Conclusions

In this study, 454 pyrosequencing provided a global understanding of the biosynthesis pathway in the non-model organism *M. incisa*. Based on the identified unique sequences and the subcellular localization of relevant proteins, a C4-like photosynthetic pathway was found to exist in *M*. *incisa* that enables this microalga to survive under low ambient CO_2_[38,68]. Nearly all unique sequences related to *de novo* ArA biosynthesis and TAG accumulation were successfully identified, thus demonstrating a more detailed lipid metabolism pathway. Furthermore, the identified unique sequences coding for PDAT, caleosin and oil globule-associated proteins helped to elucidate the mechanism of rapid accumulation and oil body formation of TAG under nitrogen starvation. In addition, the suggested carotenoid biosynthesis pathway enabled us to exploit this resource in addition to ArA production. All these unique sequences described above require functional identification prior to be used for genetically manipulating to enhance products of interest in further study.

## Methods

### Algal species and culture

The microalga *Myrmecia incisa* Reisigl H4301 was commercially provided by the Culture Collection Algae of Charles University of Prague (CAUP). A cell line isolated from this alga under a microscope was incubated in BG-11 medium in 800-mL glass flasks, which were placed in a temperature-regulated photoincubator at 25°C and illuminated from the side with a light:dark regime of 12 h:12 h using cool-white fluorescent Philips tubes (36 W) (Yizheng, Jiangsu, China) at a light irradiance of 115 μmol photons m^-2^ s^-1^[10]. During the culture, the flasks were shaken by hand several times every day at regular intervals. Algal cells were harvested with a centrifuge at 5,000 rpm in the late logarithmic growth phase and washed three times with nitrogen-free fresh BG-11 medium in which ferric ammonium citrate was substituted with ferric citrate. Subsequently, the algal cells were incubated under the mentioned conditions in fresh, nitrogen-free medium for an additional two days before harvest. The samples were washed with fresh medium without the addition of nitrogen, mixed together and stored in liquid nitrogen for total RNA extraction after centrifugation.

### RNA extraction and mRNA purification

The frozen samples were ground with a pestle and mortar in liquid nitrogen, and the total RNA was then extracted using the TRIzol reagent (Invitrogen, USA) according to the manufacturer’s protocol. Total RNA was dissolved in 200 μL of RNase-free water. The total RNA concentration was determined by NanoDrop (Thermo Scientific, USA), and the RNA integrity value was evaluated using an RNA 6000 Pico LabChip Agilent 2100 Bioanalyzer (Agilent, USA).

Total RNA was incubated with 10 units of DNase I (Ambion, USA) at 37°C for 1 h, and nuclease-free water was added to bring the sample volume to 250 μL. Messenger RNA was further purified with the MicroPoly (A) Purist Kit (Ambion, USA) following the manufacturer’s protocol. The mRNA was dissolved in 100 μL of RNA Storage Solution. The final concentration was determined using a NanoDrop.

### cDNA library construction and 454 pyrosequencing

Double-stranded cDNA was synthesized from mRNA according to Ng’s full-length cDNA synthesis protocol, with some modifications as described below [69]. A *Gsu*I-oligo dT primer was used to prime first-strand cDNA synthesis from 10 μg of mRNA using 1000 units of Superscript II reverse transcriptase (Invitrogen). After incubation at 42°C for 1 h, the 5’-CAP structure of the mRNA was oxidized by NaIO_4_ (Sigma, USA) and ligated to biotin hydrazide (Sigma, USA), which was used to select complete mRNA/cDNA by binding with Dynal M280 beads (Invitrogen). After second-strand cDNA synthesis, the poly A and 5’-adaptor was deleted by *Gsu*I digestion.

Complementary DNA size fractionation was performed using a cDNA size fractionation column (Agencourt, USA). Each cDNA fraction larger than 800 bp was sonicated to 300–800 bp and then pooled with the other cDNA samples ranging from 300 bp to 800 bp. The prepared cDNAs were transformed into single-stranded template DNA (sstDNA) libraries using the GS DNA Library Preparation kit (Roche Applied Science).

Single-stranded template DNA libraries were clonally amplified in bead-immobilized form by using the GS emPCR kit (Roche Applied Science, USA) and sequenced on the 454 Genome Sequencer FLX System (Roche Diagnostic, USA).

### Bioinformatics analysis

Using the SeqClean software (http://compbio.dfci.harvard.edu/tgi/software), low-quality (Q-value 20), low-complexity (poly A) and adaptor sequences were trimmed from the reads generated by the FLX sequencer and the qualified reads were then submitted to the CAP3 program [31] for clustering and assembly using default parameters. All unique sequences were deposited in the SRA of the National Center for Biotechnology Information (NCBI).

The open reading frame of each contig or singleton was identified using an in-house developed program based on ‘GetORF’ from EMBOSS with the parameters below: table = 0, minsize = 90 [70]. Gene annotation was performed using a BLASTp search against the Swiss-Prot and GenBank database with an E-value cutoff of 1×10^-3^ and BLASTx against the JGI Genome Portal with an E-value cutoff of 1×10^-3^ as well. The best gene annotation result was chosen by the two following principles: 1). the annotation with a clear functional description was firstly chosen; 2). the annotation with the best blast result (considering alignment coverage and identity) was chosen. Gene ontology (GO) analysis was performed using GoPipe [71] via BLASTP against the Swiss-Prot and TrEMBL databases with an E-value cutoff of 1×10^-3^. The GI accessions of the best hits were retrieved, and the GO accessions were mapped to GO terms according to the molecular function, biological process and cellular component ontologies (http://www.geneontology.org/). A metabolic pathway was constructed based on the Kyoto Encyclopedia of Genes and Genomes (KEGG) database using the BBH (bi-directional best hit) method [72]. The analysis initially retrieved the KEGG orthology (KO) number for each protein, and metabolic pathways were then constructed based on the KO number. Targeting prediction was performed using TargetP 1.1 Server (http://genome.cbs.dtu.dk/services/TargetP/) and SignalP 4.1 Server (http://genome.cbs.dtu.dk/services/SignalP/).

## Abbreviations

ArA: Arachidonic acid; PUFA: Polyunsaturated fatty acid; TAG: Triacylglycerol; DW: Dry weight; EST: Expressed sequence tag; NRS: Non-redundant sequence; HQ: High quality; GO: Gene ontology; KEGG: Kyoto encyclopedia of genes and genomes; KO: KEGG orthology; EC: Enzyme commission; RubisCO: Ribulose-1,5- bisphosphate carboxylase/oxygenase; CCM: Carbon concentrating mechanism; CA: Carbonic anhydrase; CoA: Coenzyme A.

## Competing interests

The authors declare that they have no competing interests.

## Authors’ contributions

ZGZ participated in the design of the study. SHC carried out the experiments and acquisition of original data. LLO participated in sequence analysis and she and ZGZ were involved in data interpretation and drafting the manuscript. YL assisted with the bioinformatics analysis and helped to draft the manuscript, and ZGZ gave the final approval of the version to be published. All authors have read and approved the final manuscript.

## Supplementary Material

Additional file 1 **KEGG pathway annotation of the *M*. *incisa *H4301 transcriptome.** Click here for file

Additional file 2 **Unique sequences involved in carbon concentrating mechanisms, lipid metabolism and carotenoid biosynthesis in *M***. ***incisa *H4301.** Abbreviations are listed as follows: cTP, chloroplast transit peptide; mTP, mitochondrial targeting peptide score; SP, signal peptide; RC, reliability class (1 = strong, 5 = poor prediction). Click here for file

Additional file 3 **Triacylglycerol (TAG) degradation in *M*. *incisa *H4301 based on transcriptome annotation.** The unique sequence number of each identified gene is shown in parentheses. The dashed line indicates the route that was not represented in this transcriptome. Abbreviations are listed as follows: TAGL, triacylglycerol lipase; DAGL, diacylglycerol lipase; MAGL, monoacylglycerol lipase; 2-MAG, 2-monoacylglycerol; 1-MAG, 1-monoacylglycerol; FA, fatty acid. Click here for file

Additional file 4 **Carotenoid biosynthesis model in *M*. *incisa *H4301 based on annotations in the transcriptome.** Number of the identified enzyme-coding unique sequences is shown in the flow. The dashed lines indicate the routes that were not represented in this transcriptome. Abbreviations are listed as follows: DXS, 1-deoxy-D-xylulose 5-phosphate synthase; DXR, 1- deoxy-D-xylulose 5-phosphate reductoisomerase; CMS, 2-C-methyl-D-erythritol 4-phosphate cytidyl transferase; CMK, 4-(cytidine 5’-diphospho)-2-C-methyl-D-erythritol kinase; MCS, 2-C-methyl-D-erythritol 2,4-cyclodiphosphate synthase; HDS, 1-hydroxy-2-methyl-2-(E)-butenyl 4-diphosphate synthase; HDR, 1-hydroxy-2-methyl-2-(E)-butenyl 4-diphosphate reductase; IPPi, isopentenyl diphosphate isomerase; GPPS, geranyl diphosphate synthase; FPPS, farnesyl diphosphate synthase; GGPPS, geranylgeranyl diphosphate synthase; PSY, phytoene synthase; PDS, phytoene desaturase; ZISO, zeta carotene isomerase; ZDS, zeta-carotene desaturase; CRTISO, carotenoid isomerase; β-CYC, lycopene β-cyclase; ϵ-CYC, lycopene ϵ-cyclase; BCH, β-Carotene hydroxylase; ZEP, zeaxanthin epoxidase; VDE, violaxanthin de-epoxidase; GADP, glyceraldehyde 3-phosphate; DXP, 1-deoxy-D-xylulose 5-phosphate; MEP, 2-C-methyl-D-erythritol 4-phosphate; CDP-ME, 4-(cytidine 5’-diphospho)-2-C-methyl-D-erythritol; CDP-MEP, 4-(cytidine 5’-diphospho)-2-C-methyl-D-erythritol 2-phosphate; CMEPP, 2-C-methyl-D-erythritol 2,4-cyclodiphosphate; IPP, isopentenyl diphosphate; DMAPP, dimethylallyl diphosphate; GPP, geranyl diphosphate; FPP, farnesyl diphosphate; GGPP, geranylgeranyl diphosphate. Click here for file
