# Supplementary material for: Transcriptome analysis reveals unique C4-like photosynthesis and oil body formation in an arachidonic acid-rich microalga Myrmecia incisa Reisigl H4301
Source: BMC Genomics. 2013 Jun 13;14:396. doi: 10.1186/1471-2164-14-396 (PMC3686703; doi:10.1186/1471-2164-14-396)
Supplement: Additional file 3 — Triacylglycerol (TAG) degradation in M. incisa H4301 based on transcriptome annotation. The unique sequence number of each identified gene is shown in parentheses. The dashed line indicates the route that was not represented in this transcriptome. Abbreviations are listed as follows: TAGL, triacylglycerol lipase; DAGL, diacylglycerol lipase; MAGL, monoacylglycerol lipase; 2-MAG, 2-monoacylglycerol; 1-MAG, 1-monoacylglycerol; FA, fatty acid. [file 1471-2164-14-396-S3.pdf]

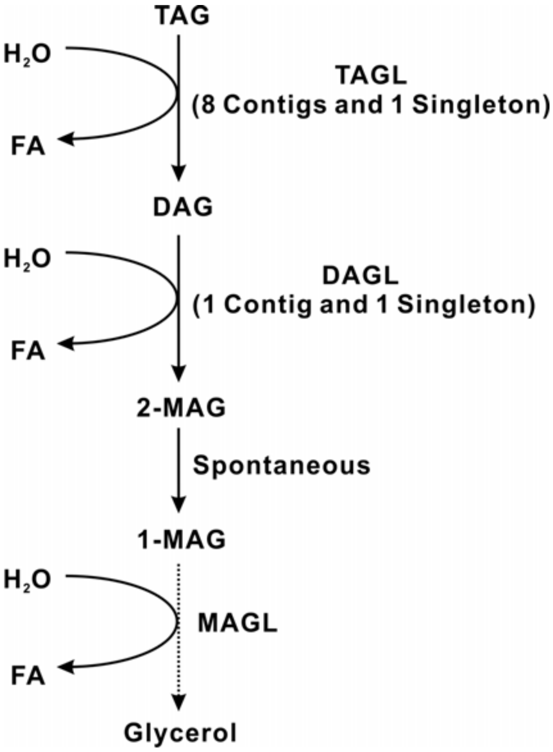

Additional file 3 Triacylglycerol (TAG) degradation model in *M. incisa* H4301 based on annotations in the transcriptome library. The number of the identified enzyme-coding unique sequences is shown in the flow. The dashed line indicates an unidentified route. Abbreviations are listed as follows: TAGL, triacylglycerol lipase; DAGL, diacylglycerol lipase; MAGL, monoacylglycerol lipase; 2-MAG, 2-monoacylglycerol; 1-MAG, 1-monoacylglycerol; FA, fatty acid.
