# Supplementary material for: Transcriptome analysis reveals unique C4-like photosynthesis and oil body formation in an arachidonic acid-rich microalga Myrmecia incisa Reisigl H4301
Source: BMC Genomics. 2013 Jun 13;14:396. doi: 10.1186/1471-2164-14-396 (PMC3686703; doi:10.1186/1471-2164-14-396)
Supplement: Additional file 4 — Carotenoid biosynthesis model in M. incisa H4301 based on annotations in the transcriptome. Number of the identified enzyme-coding unique sequences is shown in the flow. The dashed lines indicate the routes that were not represented in this transcriptome. Abbreviations are listed as follows: DXS, 1-deoxy-D-xylulose 5-phosphate synthase; DXR, 1- deoxy-D-xylulose 5-phosphate reductoisomerase; CMS, 2-C-methyl-D-erythritol 4-phosphate cytidyl transferase; CMK, 4-(cytidine 5’-diphospho)-2-C-methyl-D-erythritol kinase; MCS, 2-C-methyl-D-erythritol 2,4-cyclodiphosphate synthase; HDS, 1-hydroxy-2-methyl-2-(E)-butenyl 4-diphosphate synthase; HDR, 1-hydroxy-2-methyl-2-(E)-butenyl 4-diphosphate reductase; IPPi, isopentenyl diphosphate isomerase; GPPS, geranyl diphosphate synthase; FPPS, farnesyl diphosphate synthase; GGPPS, geranylgeranyl diphosphate synthase; PSY, phytoene synthase; PDS, phytoene desaturase; ZISO, zeta carotene isomerase; ZDS, zeta-carotene desaturase; CRTISO, carotenoid isomerase; β-CYC, lycopene β-cyclase; ϵ-CYC, lycopene ϵ-cyclase; BCH, β-Carotene hydroxylase; ZEP, zeaxanthin epoxidase; VDE, violaxanthin de-epoxidase; GADP, glyceraldehyde 3-phosphate; DXP, 1-deoxy-D-xylulose 5-phosphate; MEP, 2-C-methyl-D-erythritol 4-phosphate; CDP-ME, 4-(cytidine 5’-diphospho)-2-C-methyl-D-erythritol; CDP-MEP, 4-(cytidine 5’-diphospho)-2-C-methyl-D-erythritol 2-phosphate; CMEPP, 2-C-methyl-D-erythritol 2,4-cyclodiphosphate; IPP, isopentenyl diphosphate; DMAPP, dimethylallyl diphosphate; GPP, geranyl diphosphate; FPP, farnesyl diphosphate; GGPP, geranylgeranyl diphosphate. [file 1471-2164-14-396-S4.pdf]

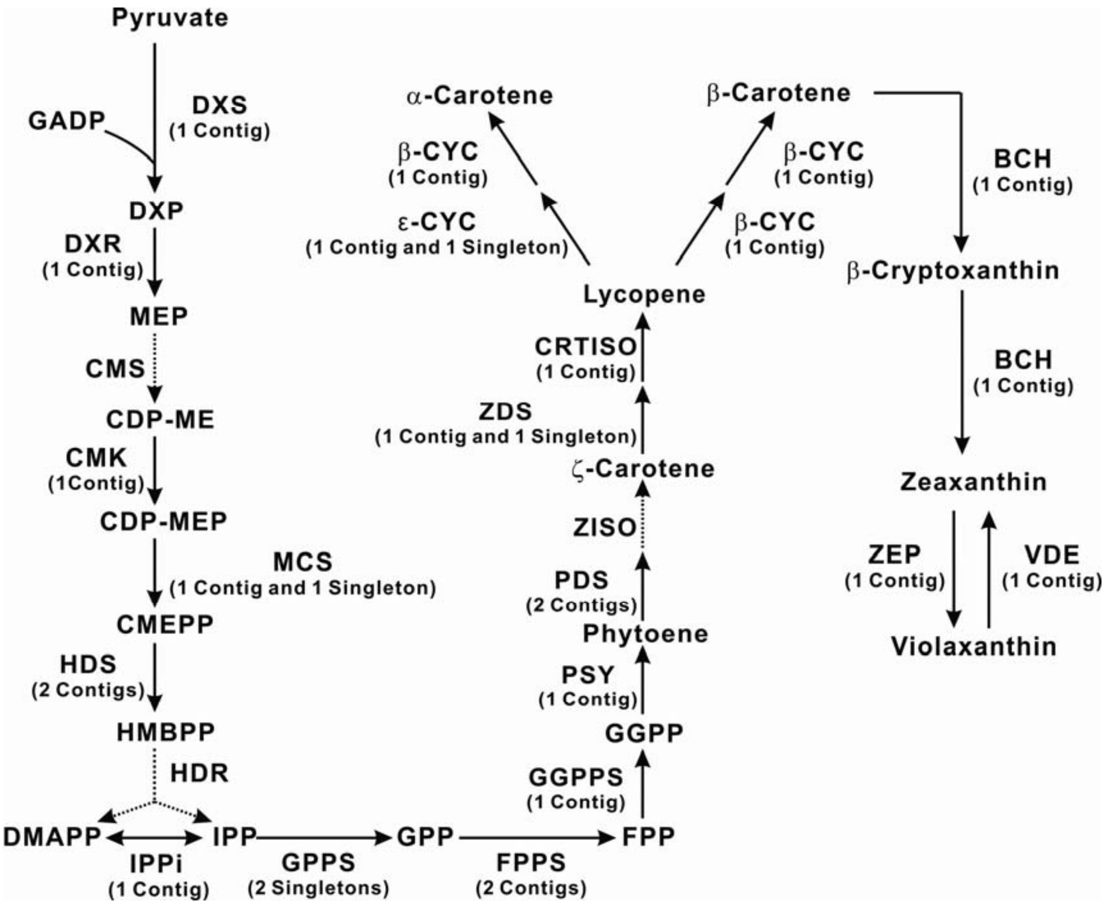

Additional file 4 Carotenoid biosynthesis model in *M. incisa* H4301 based on annotations in the transcriptome library. Number of the identified enzyme-coding unique sequences is shown in the flow. The dashed lines indicate unidentified routes. Abbreviations are listed as follows: DXS, 1-deoxy-D-xylulose 5-phosphate synthase; DXR, 1- deoxy-D-xylulose 5-phosphate reductoisomerase; CMS, 2-C-methyl-D-erythritol 4-phosphate cytidyl transferase; CMK, 4-(cytidine 5'-diphospho)-2-C-methyl-D-erythritol kinase; MCS, 2-C-methyl-D-erythritol 2,4-cyclodiphosphate synthase; HDS, 1-hydroxy-2-methyl-2-(E)-butenyl 4-diphosphate synthase; HDR, 1-hydroxy-2-methyl-2-(E)-butenyl 4-diphosphate reductase; IPPi, isopentenyl diphosphate isomerase; GPPS, geranyl diphosphate synthase; FPPS, farnesyl diphosphate synthase; GGPPS, geranylgeranyl diphosphate

synthase; PSY, phytoene synthase; PDS, phytoene desaturase; ZISO, zeta carotene isomerase; ZDS, zeta-carotene desaturase; CRTISO, carotenoid isomerase; β-CYC, lycopene β-cyclase; ε-CYC, lycopene ε-cyclase; BCH, β-Carotene hydroxylase; ZEP, zeaxanthin epoxidase; VDE, violaxanthin de-epoxidase; GADP, glyceraldehyde 3-phosphate; DXP, 1-deoxy-D-xylulose 5-phosphate; MEP, 2-C-methyl-D-erythritol 4-phosphate; CDP-ME, 4-(cytidine 5'-diphospho)-2-C-methyl-D-erythritol; CDP-MEP, 4-(cytidine 5'-diphospho)-2-C-methyl-D-erythritol 2-phosphate; CMEPP, 2-C-methyl-D-erythritol 2,4-cyclodiphosphate; IPP, isopentenyl diphosphate; DMAPP, dimethylallyl diphosphate; GPP, geranyl diphosphate; FPP, farnesyl diphosphate; GGPP, geranylgeranyl diphosphate.
